# Equecabtagene autoleucel as sequential therapy in a multiple myeloma patient with renal failure: a case report

**DOI:** 10.3389/fmed.2026.1911590

**Published:** 2026-09-23

**Authors:** Yihao Wang, Qiuying Cao, Yue Jia, Yao Zhang, Weiwei Qi, Huijuan Jiang, Jia Song, Rong Fu

**Affiliations:** Department of Hematology, General Hospital, Tianjin Medical University, Tianjin, China

**Keywords:** CAR-T cell therapy, equecabtagene autoleucel, multiple myeloma, renal failure, sequential therapy

## Abstract

This report describes the efficacy and safety of equecabtagene autoleucel (eque-cel) as sequential therapy in a multiple myeloma (MM) patient complicated with renal failure. The patient was a 50-year-old male diagnosed with MM (IgD-*λ* subtype, Durie-Salmon stage IIIB, ISS stage III, R-ISS stage II, 1q21+) with severe renal insufficiency. After 4 cycles of daratumumab, bortezomib, and dexamethasone (DVD)/daratumumab, bortezomib, pomalidomide, and dexamethasone (DVPD) induction therapy, the patient achieved hematologic complete remission (CR) and underwent autologous hematopoietic stem cell transplantation (ASCT) for consolidation. During subsequent daratumumab, ixazomib, pomalidomide, and dexamethasone (DIPD) maintenance therapy, intolerance due to recurrent infections and myelosuppression prompted a switch to eque-cel sequential therapy. During treatment, grade I cytokine release syndrome (CRS) was observed, with no severe immune effector cell–related toxicities such as immune effector cell-associated neurotoxicity syndrome (ICANS), infection, or hemophagocytic syndrome; however, hematologic toxicities including grade 3 thrombocytopenia, grade 2 neutropenia, and grade 1 anemia occurred. Efficacy evaluation at day 28 post-Chimeric antigen receptor T(CAR-T) infusion demonstrated CR with minimal residual disease (MRD) negativity. At 21 months of follow-up, the patient has not received any additional MM maintenance therapy and continues to maintain CR with MRD negativity. This case suggests that renal failure is not an absolute contraindication for CAR-T cell therapy, and that CAR-T cell therapy may serve as an alternative to novel agent therapy in selected patients who are intolerant to long-term drug treatment, yielding favorable outcomes, although further validation in more cases and prospective studies is still required.

## 1 Introduction

Multiple myeloma (MM) is a hematologic malignancy characterized by abnormal clonal proliferation of plasma cells. In the era of novel agent therapy, patient survival has improved significantly; however, long-term treatment-related toxicities frequently lead to maintenance therapy interruption or prolonged discontinuation, severely affecting patient prognosis. Chimeric antigen receptor T-cell (CAR-T) therapy, which demonstrates notable advantages in both efficacy and safety as a one-time treatment, holds an irreplaceable role in the long-term management of this currently incurable disease. At present, CAR-T cell therapy is recommended as a priority treatment for patients with relapsed/refractory or high-risk MM who have received two or more prior lines of therapy, but it is rarely used as sequential therapy after transplantation.

Renal insufficiency is one of the most common complications in MM patients. These patients have historically been excluded from CAR-T cell therapy clinical trials, particularly those with renal failure (1, 2). Our center treated one MM patient complicated with renal failure who could not tolerate novel agent maintenance therapy with eque-cel as sequential therapy. During treatment, the patient did not experience worsening of renal function, and the depth of response was further deepened, with sustained efficacy exceeding 21 months. The case is reported as follows.

## 2 Case report

A 50-year-old male was diagnosed with MM at a local hospital in February 2023 due to “foamy urine for more than 1 year.” The diagnosis was IgD-*λ* subtype, Durie-Salmon stage IIIB, ISS stage III, R-ISS stage II, with 1q21 gain, complicated by severe renal insufficiency (creatinine clearance rate 18 mL/min; baseline serum creatinine 626 μmol/L, eGFR 8.7 mL/min/1.73 m^2^, calculated using the CKD-EPI equation; 24-hour urine output approximately 1,000 mL). The patient received 2 cycles of DVD regimen and 1 cycle of DVPD regimen chemotherapy at an outside hospital, achieving CR with serum creatinine (Cr) maintained at 600–900 μmol/L. Subsequently, the patient was referred to our department for further consolidation therapy. After admission and completion of relevant examinations, an additional cycle of DVPD consolidation was administered, followed by reduced-dose melphalan (MEL140) conditioning and autologous hematopoietic stem cell transplantation (Auto-HSCT) for intensification. Hematologic response evaluation demonstrated CR with MRD positivity, and post-transplant serum creatinine was maintained at 400–600 μmol/L (Figure 1). The induction and maintenance regimens were composed as follows: the DVD regimen (daratumumab 1,100 mg on days 1, 8, 15, and 22; bortezomib 1.3 mg/m^2^ on days 1, 8, 15, and 22; and dexamethasone 20 mg on days 1–2, 8–9, 15–16, and 22–23; every 28 days as one cycle); the DVPD regimen (daratumumab 1,100 mg on days 1 and 15; bortezomib 1.3 mg/m^2^ on days 1, 8, 15, and 22; pomalidomide 3 mg on days 1–21; and dexamethasone 20 mg on days 1–2, 8–9, 15–16, and 22–23; every 28 days as one cycle); and the DIPD regimen (daratumumab 1,100 mg on days 1 and 15; ixazomib 3 mg on days 1, 8, and 15; pomalidomide 3 mg on days 1–21; and dexamethasone 20 mg on days 1–2, 8–9, 15–16, and 22–23; every 28 days as one cycle). The regimen was switched from DVD to DVPD owing to suboptimal efficacy. After ASCT, given the patient's high-risk cytogenetic abnormality of 1q21 gain (1q21+), concomitant renal insufficiency, and failure to achieve MRD negativity post-transplant, the relatively intensive DIPD regimen was selected as maintenance therapy.

**Figure 1:**
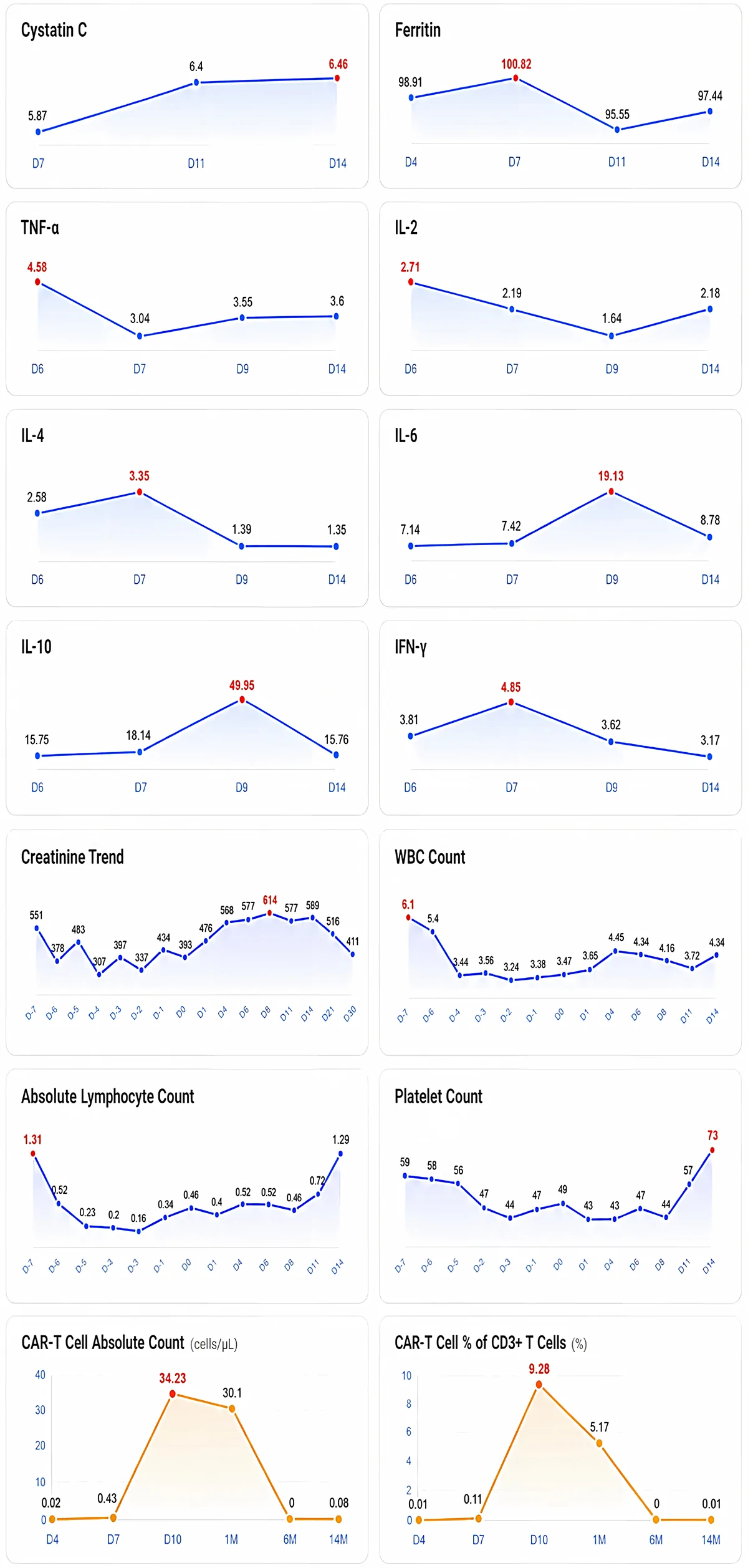
Treatment timeline of the patient. DVD = daratumumab + bortezomib + dexamethasone; DVPD = daratumumab + bortezomib + pomalidomide + dexamethasone; DIPD = daratumumab + ixazomib + pomalidomide + dexamethasone; ASCT = autologous stem cell transplantation; CAR-T = chimeric antigen receptor T cell. In this case, CAR-T cell therapy served as alternative maintenance therapy after ASCT (in the absence of salvage therapy).

Subsequently, sequential maintenance therapy with the DIPD regimen was initiated and administered for 1 cycle. During maintenance, the patient experienced recurrent herpes zoster and grade ≥3 thrombocytopenia with bone marrow toxicity, necessitating multiple hospitalizations. This not only caused repeated interruptions of maintenance therapy but also severely impaired the patient's quality of life. The patient was recommended to attempt autologous BCMA CAR-T cell therapy as sequential therapy. Given the patient's renal function status, a multidisciplinary team (MDT) consultation involving nephrology and the ICU was conducted, prophylactic clearance dialysis was performed to enhance fludarabine elimination and reduce the risk of myelosuppression, as well as to prevent lymphodepleting chemotherapy from further aggravating renal impairment, rather than as renal replacement therapy for renal failure. Under bedside hemodialysis support, lymphodepletion was performed with single-agent fludarabine (25 mg/d, approximately 15.4 mg/m^2^/d, on days 1 and 3), with dialysis administered 12 h after each dose. During this period, serum creatinine remained stable at 300–900 μmol/L, and the patient maintained adequate urine output, stable peripheral blood electrolyte levels, blood pressure, and vital signs. The dynamic changes in renal function before and after CAR-T cell infusion were as follows: with intermittent dialysis before infusion, serum creatinine was maintained at 300–400 μmol/L and remained at approximately 550 μmol/L after infusion; eGFR was maintained at approximately 15 mL/min/1.73 m^2^ before infusion and approximately 9 mL/min/1.73 m^2^ after infusion; 24-hour urine output was 1,000–1,500 mL; and serum potassium was maintained at 4–5 mmol/L before infusion and approximately 4 mmol/L thereafter. The patient did not receive further hemodialysis after CAR-T cell infusion. The lymphocyte absolute count decreased to 0.16  ×  106/L, and eque-cel (1.0  ×  106/kg) was successfully infused. Eque-cel is an autologous CAR-T cell product targeting BCMA; its CAR molecule consists of a fully human anti-BCMA single-chain variable fragment (scFv), a CD8*α* hinge and transmembrane region, a 4-1BB costimulatory domain, and a CD3*ζ* signaling domain, and the product was released after passing stringent quality control testing (3).

On day 10 post-infusion, CAR-T cells expanded to peak levels (detected by flow cytometry), accounting for 9.28% of peripheral blood CD3+ T cells with an absolute count of 34.23/μL. Cytokine levels including IL-6, IL-10, IL-2, IL-17A, IL-12, TNF-α, and IFN-*γ* (unit: pg/mL) showed no significant retention or marked elevation, and ferritin and cystatin levels remained stable (Figure 2). Following CAR-T cell infusion, the patient developed fever (37.6 °C, without other discomfort), which was considered grade I CRS. The patient developed grade 3 thrombocytopenia, grade 2 neutropenia, and grade 1 anemia. No ICANS, infection, or hemophagocytic lymphohistiocytosis (HLH) was observed.

**Figure 2:**
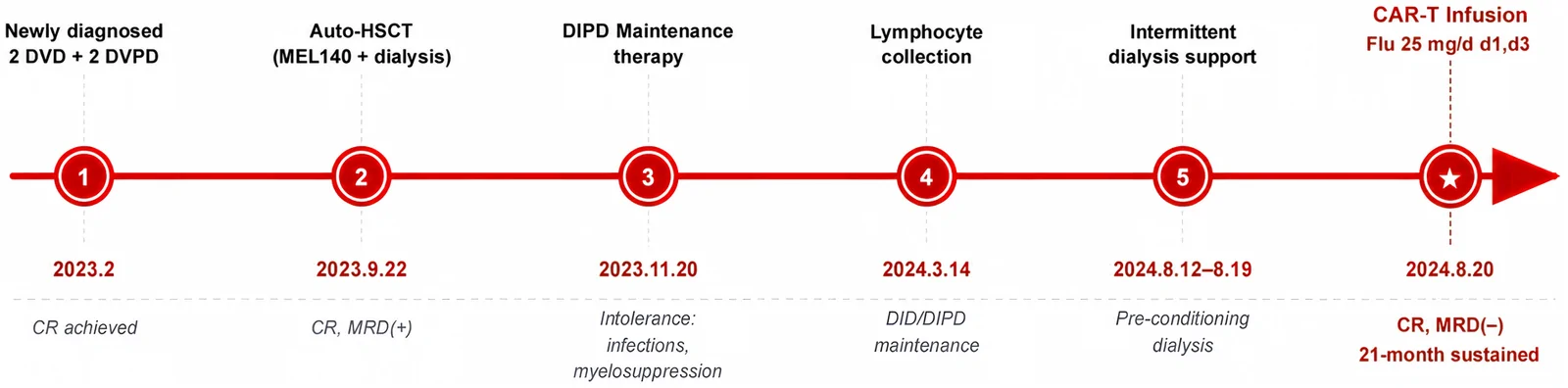
Changes in key laboratory parameters before and after CAR-T cell infusion (August 20, 2024 = day of CAR-T infusion). Panels include cystatin C, ferritin, TNF-α, IL-2, IL-4, IL-6, IL-10, IFN-*γ*, creatinine trends, white blood cell count, lymphocyte absolute count, platelet count, CAR-T cell absolute count (/μL), and CAR-T cell percentage of CD3+ T cells (%). The *x*-axis represents the time (days) relative to CAR-T cell infusion; cytokine units are pg/mL. Normal reference ranges: IL-2 0.00–5.71, IL-4 0.00–3.00 pg/mL, IL-6 0.00–5.30, IL-10 0.00–4.91, TNF-α 0.00–4.60, IFN-*γ* 0.00–7.42, ferritin 21.80–274.66 ng/mL, cystatin C 0.62–1.11 mg/L, serum creatinine 57–97 μmol/L, white blood cell count 3.50–9.50 × 10⁹/L, platelet count 125–350 × 10⁹/L, and absolute lymphocyte count 1.10–3.20 × 10⁹/L.

At the one-month post-infusion evaluation, the patient achieved complete remission (CR) with MRD negativity (Response was evaluated according to the International Myeloma Working Group (IMWG) criteria; MRD was assessed by multiparameter flow cytometry with a sensitivity of 10⁻⁵; serum immunofixation electrophoresis was negative for M protein, urine immunofixation electrophoresis was negative for M protein, the serum free light chain ratio was 0.4643, and the proportion of bone marrow plasma cells was 0%). During subsequent follow-up, the patient received no additional MM maintenance therapy. Efficacy was evaluated every 6 months. As of May 2026, at 21 months of follow-up, the patient has maintained sustained hematologic CR and MRD negativity.

## 3 Discussion

As an incurable hematologic malignancy, MM has now entered the era of immunotherapy, with significant improvements in survival and quality of life. CAR-T cell therapy, as a single-dose treatment with high remission rates, deep responses, and favorable safety profiles, is benefiting an increasing number of MM patients in clinical practice (4–7). Currently, the approved CAR-T indication in China is for relapsed or refractory or high-risk MM patients who have progressed after at least 2 prior lines of therapy, serving as a priority recommendation for salvage treatment. Few patients have received CAR-T cells as sequential therapy after intensification therapy, and there is currently insufficient evidence-based medical evidence to support this application. In recent years, BCMA-targeted CAR-T cell therapy has demonstrated definitive efficacy in relapsed/refractory MM (e.g., the KarMMa, CARTITUDE-1, and FUMANBA-1 studies) (3, 6, 7); in earlier lines of therapy, the KarMMa-3 and CARTITUDE-4 studies have confirmed that CAR-T cell therapy is superior to standard regimens (4, 5, 8), and the timing of CAR-T cell therapy continues to move forward, with preliminary explorations in frontline treatment and maintenance/consolidation therapy also reported (9).

This patient's initial treatment followed domestic and international guideline recommendations with good efficacy. However, during maintenance therapy, due to persistent renal impairment, the patient experienced significant treatment-related toxicities including recurrent viral infections and myelosuppression, leading to multiple hospitalizations for complications and interruption of maintenance therapy. This not only increased the risk of relapse and seriously affected prognosis but also substantially decreased the patient's quality of life. At present, the standard maintenance therapy for MM is lenalidomide-based (10), and daratumumab or ixazomib maintenance is also an available option (11). However, lenalidomide is primarily excreted by the kidneys, and patients with severe renal insufficiency require dose reduction and face an increased risk of myelosuppression (12); daratumumab monotherapy or ixazomib monotherapy provides relatively limited maintenance efficacy, and long-term administration still carries risks of infection and hematologic toxicity. During DIPD maintenance therapy, this patient experienced recurrent herpes zoster infection and grade ≥3 thrombocytopenia, resulting in multiple interruptions of maintenance therapy, and conventional maintenance regimens were difficult to continue. After full discussion of the benefits and risks of the available maintenance options (including DIPD regimen dose adjustment, daratumumab monotherapy, and ixazomib monotherapy), the patient chose to receive BCMA-targeted CAR-T cell therapy as sequential therapy.

During CAR-T cell therapy, lymphodepletion conditioning is required to enhance *in vivo* CAR-T cell expansion. Current lymphodepletion regimens predominantly consist of fludarabine combined with cyclophosphamide (FC) or bendamustine. For patients with renal insufficiency, both fludarabine and bendamustine carry relative or absolute contraindications. In this case, single-agent fludarabine was selected for lymphodepletion mainly based on the following considerations: fludarabine is primarily cleared by the kidneys, and drug accumulation in severe renal insufficiency can significantly increase the risk of neurotoxicity and prolonged cytopenia, so dose reduction combined with dialysis to accelerate drug clearance was required (13); cyclophosphamide and its active metabolites are also mainly excreted by the kidneys, with a high risk of cumulative toxicity in renal insufficiency, and it was therefore omitted (14); bendamustine lacks pharmacokinetic data in patients with severe renal insufficiency and can cause sustained lymphopenia and increased infection risk, and was therefore not selected (15). During lymphodepletion and after infusion, neurotoxicity and cytopenia were closely monitored, and the timing of dialysis was arranged according to drug clearance. Renal impairment is one of the most common complications in MM patients, with 20%–40% of newly diagnosed MM (NDMM) and 50% of relapsed/refractory MM (R/R MM) patients having concurrent renal impairment, and 2%–12% of NDMM patients requiring renal replacement therapy, with an even higher proportion among R/R MM patients^1,2^. Patients with renal impairment have historically been excluded from CAR-T cell therapy clinical trials; therefore, experience with CAR-T cell therapy in MM patients with renal failure, particularly those requiring dialysis, remains limited (16, 17).

We report this case of an MM patient with concurrent renal failure who, under MDT consultation and bedside dialysis support, received off-label single-agent fludarabine for conditioning. During this period, serum creatinine remained at stable levels, and eque-cel was successfully infused. Post-infusion, peripheral blood cytokines and cystatin C did not exhibit elevated levels due to retention. CAR-T cells expanded as expected, hematologic response deepened further, and MRD conversion to negative was achieved. During CAR-T treatment, no pancytopenia due to severe myelosuppression was observed., ICANS, infection, or HLH-related adverse events were observed. During the subsequent 21-month follow-up period, the patient received no additional MM-targeted maintenance therapy, and sustained efficacy was maintained. In addition, the low levels of cytokines (IL-6, IL-10, IFN-*γ*, etc.) after CAR-T cell infusion in this case were consistent with the clinical presentation of only grade I CRS. Patients with renal failure are at risk of reduced renal clearance and *in vivo* retention of cytokines and lymphodepleting agents; in this case, no obvious retention was observed under dialysis support, and neither CRS nor renal impairment was exacerbated, suggesting that reduced-dose lymphodepletion combined with CAR-T cell infusion under dialysis support is feasible in such patients.

## 4 Conclusion

As an important part of the overall management of MM, sequential therapy can significantly improve progression-free survival (PFS) and delay relapse; however, for MM patients who are intolerant to novel agents or unwilling to receive long-term drug maintenance therapy, early relapse and poor prognosis are common, and very few interventions were available in the era of conventional targeted therapy. For MM patients who are intolerant to novel agents as maintenance therapy, CAR-T cell therapy as a one-time treatment may serve as an alternative option for sequential therapy after intensification (autologous hematopoietic stem cell transplantation), with the potential to further deepen therapeutic responses. This case is an individual report of CAR-T cell therapy used as sequential therapy after autologous hematopoietic stem cell transplantation and may provide a clinical practice reference for similar patients; however, its efficacy and safety still require validation in more cases and prospective studies. In this case of an MM patient with renal impairment, under MDT and dialysis support, neither renal function nor post-infusion CAR-T cell expansion and its characteristic adverse events were severely impacted, suggesting that CAR-T cell therapy may serve as a sequential treatment option for this subset of patients. Of course, additional clinical cases and prospective studies are needed to verify the efficacy and safety of CAR-T cell therapy as sequential therapy after intensification for MM patients, including those with renal impairment.

## Data Availability

The original contributions presented in the study are included in the article/Supplementary Material, further inquiries can be directed to the corresponding author.
